# The gill parasite *Paramoeba perurans* compromises aerobic scope, swimming capacity and ion balance in Atlantic salmon

**DOI:** 10.1093/conphys/cox066

**Published:** 2017-11-29

**Authors:** Malthe Hvas, Egil Karlsbakk, Stig Mæhle, Daniel William Wright, Frode Oppedal

**Affiliations:** 1 Institute of Marine Research, 5984 Matredal, Norway; 2 Department of Biology, University of Bergen, 5020 Bergen, Norway

**Keywords:** Amoebic gill disease, U_crit_, respirometry, stress physiology, aquaculture

## Abstract

The parasitic amoeba *Paramoeba perurans* is an aetiological agent of amoebic gill disease (AGD), a serious problem in seawater salmonid aquaculture globally. Other finfish species are also infected and infection events may be associated with periods of unusual high temperatures. Currently little is known about the impact of AGD on wild fish, but in a time with global warming and increasing aquaculture production this potential threat could be on the rise. A better understanding of the pathophysiology of infected fish is therefore warranted. In this study, groups of Atlantic salmon with and without AGD were tested in a large swim tunnel respirometer in seawater at 13°C to assess oxygen uptake, swimming capacity and blood parameters. Standard metabolic rates were similar between groups, but the maximum rate of oxygen uptake was drastically reduced in infected fish, which resulted in a smaller aerobic scope (AS) of 203 mg O_2_ kg^−1^ h^−1^ compared to 406 mg O_2_ kg^−1^ h^−1^ in healthy fish. The critical swimming speed was 2.5 body lengths s^−1^ in infected fish and 3.0 body lengths s^−1^ in healthy ones. Furthermore, AGD fish had lower haematocrit and [haemoglobin], but similar condition factor compared to healthy fish. Prior to swim trials infected fish had higher plasma osmolality, elevated plasma [Na^+^], [Cl^-^] and [cortisol], indicating reduced capacity to maintain ionic homoeostasis as well as chronic stress during routine conditions. These results demonstrate that AGD compromises gill function both in terms of gas exchange and ion regulation, and consequently the capacity for aerobic activity is reduced. Reduced AS due to the *P. perurans* infections is likely to interfere with appetite, growth and overall survival, even more so in the context of a warmer and more hypoxic future.

## Teaser

A cosmopolitan non-species specific gill parasite associated with finfish aquaculture and high temperatures compromises gill functionality and swimming abilities in Atlantic salmon. Interactions with environmental warming are expected to amplify the pathophysiology of this parasite.

## Introduction

*Paramoeba perurans* is a cosmopolitan ectoparasite and an aetiological agent of amoebic gill disease (AGD); a serious problem in salmonid aquaculture globally (Munday *et al.*, 2001; Young *et al.*, 2008; Mitchell and Rodger, 2011). Although AGD in farmed Atlantic salmon has been the focus of previous studies, *P. perurans* do not elicit a clear host specificity, and currently infections in 17 species from 14 different genera has been documented (Oldham *et al.*, 2016; Kim *et al.*, 2017).

In the 1980s AGD due to *P. perurans* was mainly a problem in Tasmania during summer, but has since also occurred in salmonid aquaculture in Europe, North America, Chile, eastern Asia and South Africa (Munday *et al.* 2001; Steinum *et al.*, 2008; Mouton *et al.*, 2014; Kim *et al.*, 2016). Interestingly, infection events at new geographic locations have consistently been associated with periods of unusual high temperatures such as the first incidents in Norway and Scotland in 2006 during the warmest autumn in Europe since 1500 (Van Oldenborgh, 2007; Steinum *et al.*, 2008; Oldham *et al.*, 2016). AGD generally occurs when water temperatures reach 15–20°C (Munday *et al.*, 1990). However, more recent incidents in previously affected locations have also been reported at colder temperatures of 7–10°C (Douglas-Helders *et al.*, 2001; Rodger, 2014).

Aquaculture is increasing and in 2014 global production surpassed fisheries capture (FAO, 2016). Some aquaculture practices are associated with a range of concerns regarding sustainability and negative environmental impacts, one being pathogen release to adjacent ecosystems, where marine net pens may act as a reservoir of diseases and parasites (Murray and Peeler, 2005; Costello, 2009; Oidtmann *et al.*, 2013). Unfortunately, little is currently known about the fundamental biology and natural distribution of *P. perurans*, and its potential impact on wild fish (Stagg *et al.*, 2015; Oldham *et al.*, 2016; Hellebø *et al.*, 2017). However, given the growth of aquaculture in conjunction with climate change it can be assumed that challenges with *P. perurans* will increase in the future.

To understand and predict the consequences of anthropogenic activities on ecosystems for proper conservation management, it is important to know the physiological mechanisms of organisms under environmental change and stress (Carey, 2005; Cooke *et al.*, 2012, 2013). In particular, it is of interest to assess sublethal effects that may reduce fitness rather than mortality alone (Wood *et al.*, 2012; Cooke *et al.*, 2013). Regarding conservation of marine fish biodiversity, a useful physiological framework to integrate the effects of various environmental stressors is the ‘Fry paradigm,’ derived from the scope for aerobic activity (Fry, 1971; Claireaux and Lefrançois, 2007; McKenzie *et al.*, 2016). The aerobic scope (AS) is the difference between resting and maximum metabolic rate. It thereby provides a measurement of the available aerobic capacity to perform important life-history tasks such as foraging, growth, gonad development and locomotion (Priede, 1985; Claireaux and Lefrançois, 2007). How AS in various fish species is affected by factors such as hypoxia, elevated temperatures, ocean acidification and toxicants, and the ecological consequences thereof, is receiving tremendous attention among ecophysiologists due to climate change and other human-induced environmental impacts (Pörtner and Farrell, 2008; Clark *et al.*, 2013; Lefevre, 2016; McKenzie *et al.*, 2016). From a conservation point of view, the effects of parasitism and pathogens in fish on AS, as well as other physiological traits, and their possible interaction with abiotic stressors have received much less attention (Wagner *et al.*, 2003; Brauner *et al.*, 2012).

The clinical signs of *P. perurans* induced AGD are lethargy, anorexia and increased ventilation rates (Munday *et al.*, 1990). Pathology of the gills involves epithelial hyperplasia, lamellar fusion, mucoid lesions and necroses, which macroscopically can be observed as pale patches on the lamellae (Nowak, 2012). Since the fish gill is a multifunctional organ responsible for gas exchange, ion regulation, acid-base balance and excretion of nitrogenous waste products (Evans *et al.*, 2005), AGD has the potential to interfere with all of these crucial physiological functions.

This study was made following an unexpected AGD outbreak in several of the fish tank facilities at the Matre research station, Norway in the spring of 2017. It provided a unique opportunity to investigate the pathophysiological effects of AGD in Atlantic salmon. Swim tunnel respirometry was performed to measure the aerobic capacity and swimming ability, while also assessing effects on haematological parameters. We hypothesized that AGD would compromise gill function both in terms of gas exchange and ion homoeostasis, and thereby reduce physiological performance in infected fish.

## Materials and methods

### Animals and amoebae

Prior to experimentation, Atlantic salmon post-smolts (Aquagen, Norway) were kept in large circular tanks (5.3 m^3^) at the Institute of Marine Research in Matre, Norway for 5 months in seawater of 34 ppt under a simulated natural light regime. Water quality was ensured with a continuous open flow of 120 l min^−1^ per tank. Fish were fed commercial food pellets (Nutra, 3 mm, Skretting, Norway) in excess through automated feeding devices every day.

Infection by *P. perurans* was first suspected from white spots on the gill filaments of sampled fish, and was later confirmed with histology and PCR analyses (see below). No noteworthy mortalities or apparent change in fish behaviour in the holding facilities were observed beforehand. A series of 3 holding tanks had initially been kept at an elevated 16°C for the first 3 months, compared to a standard temperature of 9°C in the other tanks, where these warmer tanks held the most severely infected fish. For the following 2 months after initial temperature acclimation, all holding tanks were maintained at an identical temperature of 13°C. Once AGD was confirmed, a 4 h fresh-water bath was given the following day to all fish at the research station as a treatment for AGD (Munday *et al.*, 1990; Powell *et al.*, 2015), except for one of the holding tanks with the previously warm-acclimated fish.

Experiments were conducted with ethical approval abiding by Norwegian laws and regulations for procedures on live animals under permit number 9776.

### Experimental protocols

A custom-built large Brett-type swim tunnel respirometer, designed to assess natural swimming behaviours in groups of fish was used in this study as previously described (Remen *et al.*, 2016; Hvas *et al.*, 2017). Briefly, the swim section of the tunnel was 248 cm long and had an internal diameter of 36 cm, with easy access at the rear end through a removable lid. An O_2_ sensor was deployed behind the rear grid of the swim section that logged O_2_ concentration in the water every two seconds (RINKO ARO-FT, JFE Advanced Co., Japan). In the same location, a camera was placed to observe undisturbed fish behaviour. Controlled water currents were generated with a motor-driven propeller (Xylem Solutions, Norway) and a frequency converter (ITT Monitoring and Control, Norway). The system was connected to a temperature regulated water reservoir (13°C) via a wide hose, which allowed for controlled flushing during swim trials to maintain temperature and oxygen levels.

Groups of 15 fish were gently netted from the holding tanks and quickly moved to the tunnel, where they were allowed to acclimate over night at 20 cm s^−1^ before the swim challenge commenced. The swim challenge was a typical critical swimming speed (U_crit_) protocol (Brett, 1964; Plaut, 2001), where the water current velocity increased stepwise by 10 cm s^−1^ every 20 min until all fish reached fatigue, defined as when they became unwilling to continue swimming even with tactile stimulation.

When fatigued, individual fish were quickly removed from the swim tunnel and stunned with a blow to the head. A 3 ml blood sample was immediately drawn from the caudal vein with a heparinized syringe and momentarily stored on ice. Each gill arch was then thoroughly inspected and scored on a scale from 0 to 5, with 5 representing a severe AGD infection and 0 representing no signs of infection (Adams *et al.*, 2004; Taylor *et al.*, 2009; Bridle *et al.*, 2010). A gill tissue piece from the second left gill arch was dissected out and frozen at −80°C for real-time RT-PCR analysis for *P. perurans*. For histology, the second right gill arch from the two worst and two best swimmers in each swim trial was dissected out and stored in formalin. Weight (W) and fork length (L_f_) was then recorded.

Fish with AGD were tested in the same week the outbreak was discovered, while normal fish were tested 4 weeks following the fresh-water treatment. Four replicated swim trials were performed both with infected and non-infected groups. In addition, blood samples were drawn from infected and non-infected salmon netted directly from the holding tanks and anaesthetized in Finquel MS-222 (10 mg l^−1^), to provide control haematological parameters from individuals that did not fatigue in the swim tunnel (*N* = 20).

### Swim tunnel respirometry

During swim trials the system was kept closed for 15 min and subsequently flushed for 5 min to restore oxygen levels at every velocity interval. Oxygen consumption rates (MO_2_) were then calculated from the linear decrease in oxygen concentration as a function of time for each closed period (Steffensen, 1989; Hvas *et al.*, 2017). The resting metabolic rate, termed standard metabolic rate (SMR) in fish and other ectotherms, was estimated by fitting an exponential regression to MO_2_ as a function of swimming speed and back-extrapolating to a swimming speed of 0 (Brett, 1964; Beamish, 1978). The highest measured MO_2_, the maximum metabolic rate (MMR), coincided with the swimming speed where fish reached fatigue. AS was then calculated as MMR—SMR.

Swimming performance, expressed as U_crit_, was calculated according to Brett (1964) as the last completed swimming speed plus the proportion of the time interval endured where fatigue was reached multiplied by the velocity increment. Solid blocking effects were not corrected for, since individual fish did not exceed 10% of the tunnel cross-sectional area and rarely overlapped when swimming during trials (Bell and Terhune, 1970; Plaut, 2001; Hvas *et al.*, 2017).

### Blood parameters

After blood sampling the haematocrit (Hct) was measured in duplicates as the red blood cell fraction in capillary tubes after spinning for 2 min in a standard haematocrit centrifuge (StatSpin MP Centrifuge). Simultaneously 2 ml blood was centrifuged at 3000 g for 5 min to obtain blood plasma, which was stored in Eppendorf tubes at −80°C for subsequent analyses. Following these procedures, the haemoglobin concentration (Hb) was measured with a Hb assay kit (MAK115, Sigma-Aldrich).

Plasma Na^+^, K^+^ and Cl^−^ concentrations were measured with a Cobas 9180 electrolyte analyser (Roche Diagnostics). Osmolality was measured with a Fiske 210 Micro-Sample Osmometer (Advanced Instruments). Plasma lactate concentrations were measured spectrophotometrically with MaxMat PL (MaxMat). Cortisol was quantified with an ELISA assay kit (IBL International GmbH) and a Sunrise microplate reader (Tecan).

### Histology and qPCR analyses

Formalin fixed gill samples were embedded in paraffin according to standard protocols. Paraffin sections (3 μm) were then stained with Masson’s HES (Hematoxylin-erythrosine-saffron) stain.

Total RNA was extracted from gill tissue with Promega Reliaprep simplyRNA HT 384, art nr ×9601(Nerliens) on a Biomek 4000 Laboratory Automated Workstation (Beckman Coulter) according to the manufacturer’s instructions, and quantitated using a NanoDrop™ 1000 spectrophotometer (Thermo Scientific). The RNA samples were normalized to the concentration of 100 ng μl^−1^ using the Biomek 4000 Laboratory Automated Workstation (Beckman Coulter). Genomic DNA was extracted from gill tissue with Qiagen DNeasy 96 Blood&Tissue Kit (12), art nr 69 582(Qiagen) according to the manufacturer’s instructions, and quantitated using a NanoDrop™−1000 spectrophotometer. The DNA samples was normalized to the concentration of 100 ng/μl.

The qPCR for *P. perurans* was conducted using the assay designed to amplify a part of the 18S rRNA gene of the amoeba (Fringuelli *et al.* 2012). The qPCR was performed using AgPath-ID One Step RT-PCR reagents, art nr 4 387 424(Thermo fisher) according to the manufacturer’s instructions. The elongation factor 1α gene (ELF) of Atlantic salmon was used as endogenous control (Olsvik *et al.*, 2005). During histological examination signs corresponding to salmon gill poxvirus (SGPV) infection was seen in some fish. Therefore, extracted DNA was screened using qPCR also for (SGPV) according to the instructions published by Gjessing *et al.* (2015). The qPCR assay was run using TaqMan™ Fast Universal PCR Master Mix (2X), no AmpErase™ UNG Catalog number 4 352042 (Life Technologies AS) according to the manufacturer’s instructions, with 2 μl of normalized gill DNA in a reaction mix containing 900 nM of forward primer, 900 nM of reverse primer and 250 nM of probe in a total volume of 10 μl on 384 well-plate.

For the qPCR assay, amplification and fluorescence detection were performed by a 7900HT Fast Real-Time PCR system (Applied Biosystems) for 40 cycles with threshold values of 0.2 (*P. perurans* and ELF) or 0.02 (SGPV).

### Statistics

Differences in measured parameters between AGD and normal fish were tested for with a *t*-test or with the Mann–Whitney rank sum test when normality could not be confirmed with the Shapiro–Wilk test. This was done either between the two groups from the swim trials or the two controls groups. The strength of correlation between amoeba load and swimming performance in individual infected fish were assessed with the Pearson correlation coefficient. All analyses were made with Sigmaplot 12.3, Systat Software. The level of significance was set to 0.05. Data are presented as means ± s.e.m unless specified otherwise.

## Results

### Fish health

**Figure 1:**
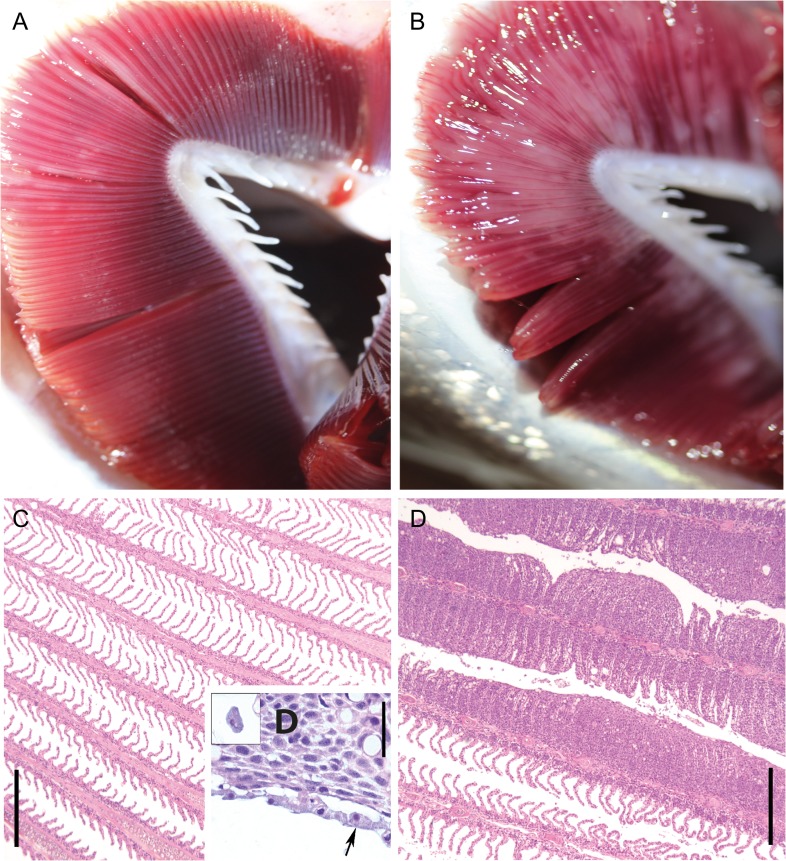
Representative picture of normal (**A**) and *Paramoeba perurans* infected (**B**) gill arch, with gill scores of 0 and 5, respectively. Representative histology of normal (**C**) and *P. perurans* infected (**D**,**E**) gills. Affected gill filaments showed massive interlamellar hyperplasia associated with the amoebae (D,E), the amoebae occurring as a layer on top of the epithelium (D, arrows), or seen interlamellar (inserted). Bars, C,E 300 μm, D, 30 μm.

The severity of AGD was highly similar within the infected group with gill scores of 4.1 ± 0.1, while no signs of AGD were observed in normal fish (gill score of 0). Two AGD fish died overnight after transfer to the swim tunnel, which reduced this group to 58 individuals. Gill histology in infected fish revealed extensive epithelial hyperplasia, lamellar fusion and associated amoebae (Fig. 1D and E). SGPV was only detected in 5 fish (high C_t_ values of 27–37), all in the AGD group, and was therefore considered negligible. The real-time RT-PCR analyses confirmed the presence of *P. perurans* in all fish tested (100% prevalence, *N* = 58) from the untreated holding tanks, with C_t_ values of 12.3–18.6 (mean of 15.6 ± 0.2). W, L_f_ and K were similar between infected and non-infected fish, while infected fish had lower Hct and Hb (Table 1). The Hct/Hb ratio was similar between groups, indicating that differences in Hct were not caused by osmotic volume change of erythrocytes.

**Table 1:** Weight (W), fork length (L_f_), condition factor (K), haematocrit (Hct) and [haemoglobin] (Hb) in the experimental groups. *N* = 58 for AGD_fatigue_, *N* = 60 for Normal_fatigue_ and *N* = 20 for control groups. Asterisks indicate a significant difference within fatigue or control groups. Data are means ± s.e.m.

	W (g)	L_f_ (cm)	K	Hct (%)	Hb (mM)
AGD_fatigue_	335 ± 9	31.3 ± 0.3	1.08 ± 0.01	33.5 ± 1.3*	1.25 ± 0.04*
Normal_fatigue_	347 ± 8	31.7 ± 0.3	1.08 ± 0.01	40.0 ± 0.6	1.52 ± 0.03
AGD_control_	324 ± 13	31.1 ± 0.4	1.07 ± 0.02	35.0 ± 1.1*	1.26 ± 0.06*
Normal_control_	330 ± 15	31.0 ± 0.5	1.09 ± 0.01	41.6 ± 0.7	1.48 ± 0.08

### Aerobic capacity and swimming performance

**Figure 2:**
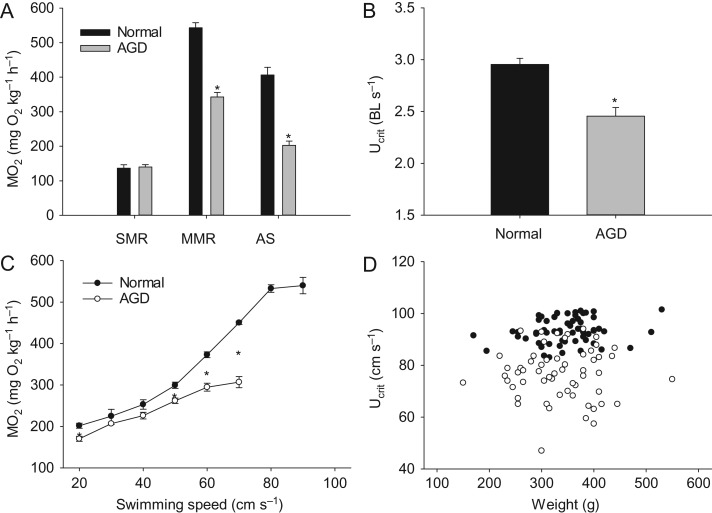
Standard metabolic rate (SMR), active metabolic rate (AMR) and aerobic scope (AS) (**A**), critical swimming speed (U_crit_) in body lengths s^−1^ (**B**), oxygen uptake rate (MO_2_) as a function of swimming speed (**C**), and scatter plot of U_crit_ in cm s^−1^ versus weight in individual fish (**D**) in infected (open symbols) and non-infected (closed symbols) fish. Statistical differences are indicates with asterisks. Data are means ± s.e.m.

Although SMR was similar between groups, MMR was drastically reduced in AGD fish, which resulted in a much lower AS of 203 ± 12 mg O_2_ kg^−1^ h^−1^ compared to 406 ± 22 mg O_2_ kg^−1^ h^−1^ in non-infected fish (Fig. 2A).

**Figure 3:**
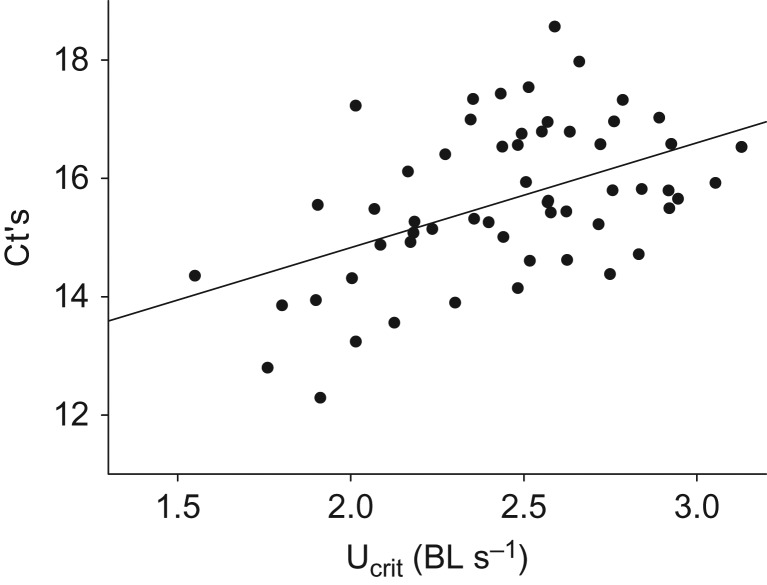
Individual amoebae load (as C_t_) versus the critical swimming speed (U_crit_) in infected Atlantic salmon (*r* = 0.50, *N* = 58, *P* < 0.001). Note that increasing C_t_ values correspond to fewer amoebae, meaning that U_crit_ tend to decrease with increasing amoeba density.

A slightly, but significantly lower MO_2_ in the AGD group at the initial acclimation speed suggested less spontaneous activity in calm conditions for infected fish. At the following increments at moderate speeds with steady swimming, MO_2_ was similar between groups. However, at 50 cm s^−1^ and above AGD fish seemed unable to meet the increasingly higher oxygen requirements to sustain aerobic swimming compared to normal fish (Fig. 2C). As a consequence, U_crit_ was reduced from 2.96 ± 0.06 body lengths s^−1^ in normal fish to 2.46 ± 0.08 body lengths s^−1^ in AGD fish (Fig. 2B). A scatter plot of U_crit_ versus W of individual fish showed that the size distribution was similar between the two groups, and that U_crit_ remained independent of W within the limited size range tested for here, while fish with and without AGD were clearly separated into different clusters based on swimming performance (Fig. 2D). Within the AGD group, there was a highly significant positive correlation between U_crit_ and C_t_ values (*r* = −0.50, *N* = 58, *P* < 0.001), such that more infected individuals tended to elicit a poorer swimming performance (Fig. 3).

### Plasma ions

**Figure 4:**
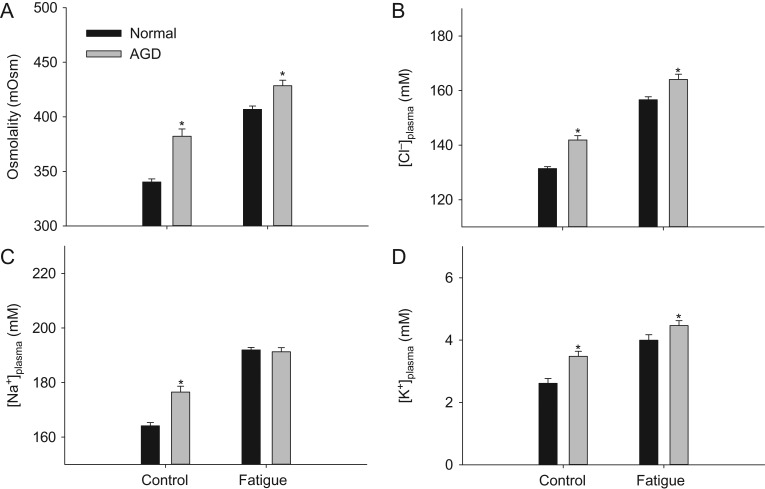
Plasma osmolality (**A**), [Cl^-^] (**B**), [Na^+^] (**C**) and [K^+^] (**D**) in fish with and without AGD. Statistical differences are indicates with asterisks. Data are means ± s.e.m.

Swimming to exhaustion caused a large osmotic disturbance with elevated [plasma ions] regardless of infection status (Fig. 4A–D). In specimens not tested in the swim tunnel, AGD fish showed higher osmolality and higher plasma concentrations of Cl^−^, Na^+^ and K^+^ compared to non-infected fish, indicating that AGD caused problems with maintaining ionic homoeostasis even during routine conditions. Furthermore, Osmolality, [Cl^−^] and [K^+^] remained significantly elevated after swimming to exhaustion in infected fish, while [Na^+^] were similar to non-infected fish when fatigued.

### Stress parameters

**Figure 5:**
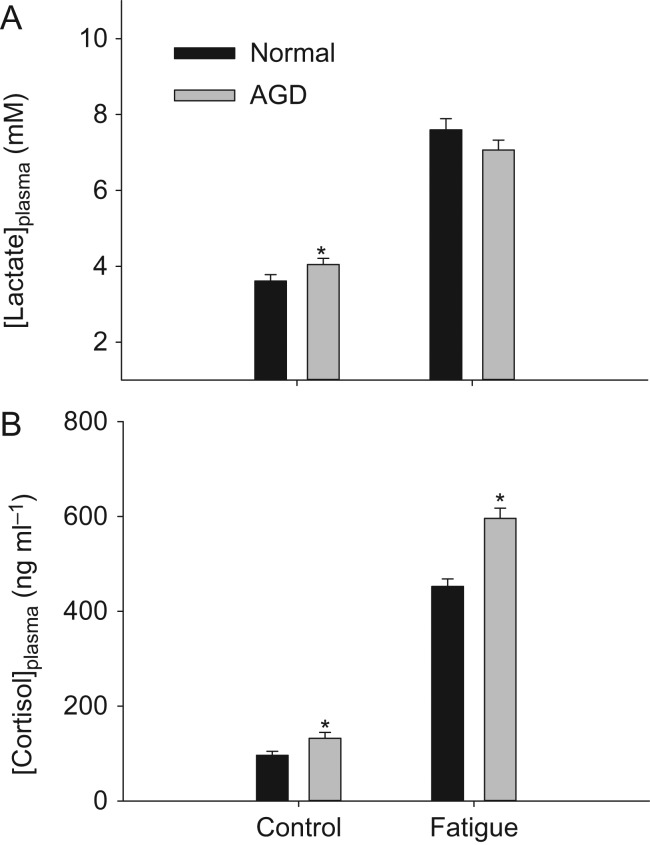
Plasma concentrations of lactate (A) and cortisol (**B)** in fish with and without AGD. Statistical differences are indicated with asterisks. Data are means ± s.e.m.

Plasma [lactate] was similar between infected and non-infected fish after swim trials, indicating equal levels of physiological exhaustion. In control conditions, AGD fish showed elevated plasma [lactate] compared to normal fish (Fig. 5A). Plasma [Cortisol] was higher in fish with AGD, both in control conditions and after swim trials (Fig. 5B).

## Discussion

### Physiological consequences of AGD

Numerous physiological disturbances were observed in Atlantic salmon with AGD owing to *P. perurans*. Most important was a drastic reduction in oxygen uptake during high-intensity swimming, which effectively cut AS in half and impaired swimming performance. The limit of MMR is defined by the capacity of the cardiovascular and respiratory systems in uptake, transport and delivery of oxygen to the receiving respiring tissues (Gallaugher *et al.*, 2001; Wang and Malte, 2011; Norin and Clark, 2016). Several anatomical, physiological and biochemical components may in theory modulate this capacity. However, when considering the pathology of AGD, the most proximal cause for a dramatic reduction in MMR as found here is impairment of gill function, presumably from a reduction in functional surface area and increased diffusion distance in the water-blood barrier. Reduction in functional gill surface area has previously been shown to decrease MMR and U_crit_ in salmonids (Duthie and Hughes, 1987).

Anaemia also reduces MMR and U_crit_ in salmonids (Jones, 1971; Gallaugher *et al.*, 1995). Other ectoparasites such as *Lepeophtheirus salmonis* may cause anaemia and reduce U_crit_ (Wagner *et al.*, 2003; Wagner and McKinley, 2004). Hence, in the present study a Hct of 33.4% in fish with AGD compared to 40.0% in normal fish may have contributed to the impairment of aerobic capacity. However, a Hct of 33.4% is still well within the normal range for salmonids, while anaemia generally is defined as <20% (Gallaugher *et al.*, 1995; Wagner and McKinley, 2004). The drastic reduction in MMR is therefore likely to primarily be caused by damage of the gill tissues.

One previous study has also attempted to quantify AS in Atlantic salmon with AGD, where it surprisingly reported that it was unaffected over an experimentally-induced AGD infection period (Leef *et al.*, 2007). In that particular study, flow-through respirometry was used, while MMR was measured after a chase protocol with a 25-min delay. As opposed to intermittent-flow respirometry, open flow respirometry poses several issues and is generally not recommended, especially during non-steady state conditions (Steffensen, 1987). Furthermore, as opposed to the U_crit_ test, a chase protocol may underestimate MMR in fish with good swimming capabilities such as Atlantic salmon (Norin and Clark, 2016). Hence, Leef *et al.* (2007) reported MMR of 125 g healthy Atlantic salmon at 16°C to be ~350 mg O_2_ kg^−1^ h^−1^, while MMR values of ~500 mg O_2_ kg^−1^ h^−1^ have been obtained in 300–500 g Atlantic salmon at 9–13°C with intermittent-flow swim tunnel respirometry (Wilson *et al.*, 2007; Hvas *et al.*, 2017; the present study). Compensating for size and temperature effects would magnify these substantial differences even further. A reduction in AS may therefore not have been observed previously by Leef *et al.* (2007) owing to inadequate methodology.

During routine conditions, infected fish showed elevated concentrations of plasma ions and cortisol, which suggests they were chronically stressed and struggled to maintain osmotic homoeostasis. Chronic stress in conjunction with elevated plasma chloride has also been documented in salmonids infected with the ectoparasite *Lepeophtheirus salmonis* (Bjørn *et al.*, 2001; Wagner *et al.*, 2003). A cortisol mediated stress response increases the permeability of the surface epithelia of the gills which increases gas exchange and ion uptake (Wendelaar Bonga, 1997). Furthermore, as a multifunctional organ the gill is subject to an osmorespiratory compromise in any physiologically demanding situation (Wood and Randall, 1973; Nilsson and Sundin, 1998). Therefore, given the presumably drastic reduction in functional gill area, infected fish were likely facing an imbalance in oxygen requirements and ion regulation, which also was evident from higher plasma lactate. As a consequence, a shift in osmotic homoeostasis occurred in infected fish during routine conditions.

Regardless of infection status, exercising to fatigue increased cortisol levels by a factor of ~4.5, and was accompanied by elevated plasma lactate and osmolality. These are expected physiological responses to an intensive swim challenge in salmonids (Brauner *et al.*, 1992; Wagner *et al.*, 2003). Interestingly, the capacity for anaerobic metabolism in infected fish appeared unaffected, since plasma lactate concentrations were similar to that of normal fish after reaching fatigue. In the swim trials, infected fish attained a higher osmolality, namely due to higher plasma chloride concentrations. Hence, the absolute disturbance in routine ion balance was more severe in fish with AGD after strenuous exercise. Recovery metabolism was not assessed in this study, but fish with AGD would likely require significantly more time to restore osmorespiratory balance, and therefore be more prone to rapid exhaustion in repeated swim challenges.

Evidently, the physiological consequence of AGD involves both a dramatic reduction in the capacity for oxygen uptake and a disturbance in ion balance, which impairs aerobic swimming performance. Unsurprisingly, the proximal pathological effects reported here are, therefore, ultimately all associated with reduced gill function. Furthermore, in the long-term, chronic stress from elevated cortisol may also interfere with immune responses and growth (Wendelaar Bonga, 1997).

### Severity of gill infection and its ecological relevance

The severity of gill pathology in this study was assessed with a predefined scoring system, which was originally developed as a quick and robust on-site indicator of disease level for management specific purposes in aquaculture (Adams *et al.*, 2004; Taylor *et al.*, 2009; Bridle *et al.*, 2010). All infected fish scored high on this scale, meaning that the ecological relevance of the present study, in theory, could be disputed if it is assumed that they inevitably would die soon if left untreated. However, this scoring system is not a precise measurement of the proportion of gill damage, and the types of changes are only classified when visible to an observer. Scores may be influenced by the individual examiner and the available time per fish in amongst routine practices on a fish farm. Furthermore, anecdotal reports suggests that gill scores tends to be lower outside in ambient light conditions compared to in a well-lit laboratory setting (Pers. Comm. Daniel Wright). The gill scores reported here may therefore be higher compared to field work observations on similarly infected fish. Unfortunately, this makes it difficult to accurately compare the severity of AGD between outbreaks, while a precise measure of percentage gill area affected would be a tremendous task requiring time consuming techniques such as histology and stereology. A compromise in future studies for representative measure of infection level would be to still score each gill, but with measurements rather than rankings of the degree of changes. This could be done on images of fresh or fixed gills (Adams *et al.*, 2004).

The fish studied here were gill scored after the performance testing meaning that the severity of infection was not known initially. In farms, the monitoring of health status should ideally not allow such high scores to develop, and fish would then have been treated at an earlier stage. Therefore, it is highly relevant to also test a moderately infected group (e.g. gill scores of 2–3) for a more nuanced perspective of the pathophysiology of AGD. However, based on our current knowledge, the sheer magnitude of the observed reduction in physiological capabilities induced by *P. perurans*, including AS being cut in half, it can be inferred that a more moderate infection status also must compromise physiological performance.

Interestingly, in light of the apparent severity of infection it is perhaps perplexing that the outbreak was not discovered earlier given the fact that fish were kept in a carefully monitored research facility with daily inspections by an experienced technical staff. However, clinical symptoms such as lethargy, anorexia, poor K-factor and mortality were not observed prior to experimentation, even though amoeba likely had been present in the tank systems for a long time. Infected fish were clearly able to meet their routine oxygen requirements, and it was therefore not until an exhaustive swim challenge that the dramatic reduction in physiological capacity was revealed. The pathophysiological effects reported here can therefore be considered relevant in an ecological context.

To survive and compete in a natural setting, good exercise capabilities are crucial (Brauner *et al.*, 2012). Fish with reduced AS owing to AGD are, therefore, more likely to be eaten by predators, less likely to catch prey, and will in addition be disadvantaged when performing tasks with a substantial aerobic requirement such as digestion, gonad development and migration.

*P. perurans* has so far been documented in at least 17 fish species (Oldham *et al.*, 2016; Kim *et al.*, 2017), however, little is currently known about the pathological consequence in non-salmonids or whether certain species are more susceptible to AGD compared to others. If wild fish develop disease, this may be difficult to observe since infected fish are less likely to survive and get caught by a researcher. Attempts to assess AGD abundance on various wild fish species including in the proximity of AGD affected salmon farms have been done in Tasmania, Scotland and Norway, but few have been found infected (Douglas-Helders *et al.*, 2002; Hjeltnes *et al.*, 2014; Stagg *et al.*, 2015; Hellebø *et al.*, 2017). However, some sampling techniques such as line and reel or fish traps, as used in the study by Douglas-Helders *et al.* (2002) and Hellebø *et al.* (2017), respectively, are likely to select against potential infected fish showing lethargic and anorexic behaviours. Hence, while AGD has the potential to severely compromise fitness-related traits it remains to be elucidated whether an ecological impact is being made on wild fish.

### Interactions with abiotic factors

Hypoxia has become an increasing threat to marine ecosystems globally because of anthropogenic eutrophication from nutrient runoff (Diaz and Rosenberg, 2008; Friedrich *et al.*, 2014), while climate change causes ocean temperatures to increase (IPCC, 2014). Much is already known about how these abiotic factors affect the physiology of Atlantic salmon as well as other salmonid fish. It is therefore relevant to briefly discuss some of their possible interactions with AGD.

Moderate environmental hypoxia reduces U_crit_ and MMR in salmonids, while SMR is unaffected (Jones, 1971; Wood *et al.*, 2017). Meeting MO_2_ requirements will therefore first become a problem during exercise, similarly to fish with AGD. Consequently, infected fish in hypoxia will have even less aerobic capacity available to perform activities.

Elevated temperatures drastically increases MO_2_, although AS is preserved within ecologically relevant temperatures in Atlantic salmon. However, owing to the increase in SMR, the factorial AS (MMR/SMR) is reduced at higher temperatures (Hvas *et al.*, 2017). A low factorial AS may compromise appetite and digestion (Farrell, 2016), while higher temperatures increases the metabolic costs of living which needs to be compensated by an increased feed intake (Morgan *et al.*, 2001). Hence, at higher temperatures factorial AS will be further reduced in fish with AGD which likely will interfere with appetite, the capacity for digestion, and consequently impair growth. This may explain why anorexia and lethargy often are reported as clinical symptoms of AGD occurring at 16–20°C, and explain why these symptoms were not observed in the present study at 13°C where the factorial AS presumably still was sufficient to support growth in Atlantic salmon.

Finally, with reduced functional gill area, an increase in ventilation will be required for similar gas transfer rates compared to fish with healthy gills. Both hypoxia and elevated temperatures will increase relative ventilation requirements in infected fish owing to less oxygen per volume ventilated and higher metabolic rates, respectively. Such increases in ventilation may lead to higher rates of amoeba transmission and hence further increase the risks of parasitism (Mikheev *et al.*, 2014).

### The role of pathogens in studies on fish conservation physiology

With the widespread use of the Fry paradigm as a central integrator in understanding the ecophysiology of fish for conservations purposes (McKenzie *et al.*, 2016), the possible metabolic effects of pathogens appears underappreciated in the literature. Here we show that one such pathogen, *P. perurans*, reduces AS, U_crit_ and shifts osmotic homoeostasis in Atlantic salmon, and points to knowledge gaps in the potential ecological impact of this cosmopolitan parasite that show low host specificity. In addition, other parasites may affect condition and reproduction of fish (Ferrer-Maza *et al.*, 2016), as well as other physiological functions such as obstructing blood flow (Coleman, 1993), and impair muscle function by encysting in the musculature (Butler and Millemann, 1971; Sprengel and Lüchtenberg, 1991).

In amphibian conservation biology it is already well-established that pathogens and climate change synergistically causes population declines and extinctions, and that risk assessments focusing only on a single stressor are likely to reach a more optimistic conclusion (Pounds *et al.*, 2006; Hof *et al.*, 2011). Thus, while abiotic factors such as hypoxia and temperature are important physiological drivers in fish ecology, perhaps synergistic effects with pathogens such as *P. perurans* during environmental change could be the deciding factor in shaping fish populations in the future.
